# Data processing and classification analysis of proteomic changes: a case study of oil pollution in the mussel, *Mytilus edulis*

**DOI:** 10.1186/1477-5956-4-17

**Published:** 2006-09-13

**Authors:** Tiphaine Monsinjon, Odd Ketil Andersen, François Leboulenger, Thomas Knigge

**Affiliations:** 1 IRIS – International Research Institute of Stavanger AS, Randaberg, Norway; 2 Laboratoire d'Ecotoxicologie – Milieux Aquatiques, Université du Havre, Le Havre, France

## Abstract

**Background:**

Proteomics may help to detect subtle pollution-related changes, such as responses to mixture pollution at low concentrations, where clear signs of toxicity are absent. The challenges associated with the analysis of large-scale multivariate proteomic datasets have been widely discussed in medical research and biomarker discovery. This concept has been introduced to ecotoxicology only recently, so data processing and classification analysis need to be refined before they can be readily applied in biomarker discovery and monitoring studies.

**Results:**

Data sets obtained from a case study of oil pollution in the Blue mussel were investigated for differential protein expression by retentate chromatography-mass spectrometry and decision tree classification. Different tissues and different settings were used to evaluate classifiers towards their discriminatory power. It was found that, due the intrinsic variability of the data sets, reliable classification of unknown samples could only be achieved on a broad statistical basis (n > 60) with the observed expression changes comprising high statistical significance and sufficient amplitude. The application of stringent criteria to guard against overfitting of the models eventually allowed satisfactory classification for only one of the investigated data sets and settings.

**Conclusion:**

Machine learning techniques provide a promising approach to process and extract informative expression signatures from high-dimensional mass-spectrometry data. Even though characterisation of the proteins forming the expression signatures would be ideal, knowledge of the specific proteins is not mandatory for effective class discrimination. This may constitute a new biomarker approach in ecotoxicology, where working with organisms, which do not have sequenced genomes render protein identification by database searching problematic. However, data processing has to be critically evaluated and statistical constraints have to be considered before supervised classification algorithms are employed.

## Background

In ecotoxicology, proteomics has been increasingly applied for the screening of protein expression changes caused by pollutants [1-10]. Proteomic profiles are generally altered upon stress and hence should also be distinctive of certain types of toxic exposure [2]. 2-DE is still the prevailing tool for protein separation and analysis, followed by MS to identify the proteins of interest. MS-based protein profiling is rapidly emerging within the current applications of proteomic technologies. Especially in medical research, it has received increasing attention for the development of new diagnostic criteria and disease monitoring. Consequently, there exists a large body of literature on how to best exploit datasets from proteomic MS, comprising large sample sizes and high dimensionality. Owing to the amount and complexity of the generated data, machine-learning techniques have been considered the methods of choice for analysing such type of multivariate data [11,12]. A plethora of supervised learning methods, such as partial least squares, discriminant and logistic regression analysis, genetic algorithms, artificial neural networks, *k*-nearest-neighbour, support vector machines and decision trees have been evaluated for this purpose [reviewed in [11,13,14]].

In conjunction with SELDI, this concept has also been introduced into ecotoxicology [5]. After Hogstrand *et al*. employed SELDI for protein profiling of rainbow trout gills during exposure to waterborne zinc [2], Knigge *et al*. have suggested the use of classifiers based on ProteinChip mass-spectra to identify field-exposure to copper or PAH in the bivalve *Mytilus edulis *(L.) [5], widely used as an environmental sentinel. Such classifiers may constitute a so-called "biomarker pattern" which can be of high discriminatory power, regardless of the identity of the specific proteins that form it.

The global analysis of cellular constituents potentially provides a more comprehensive view of toxicity, since toxicity generally involves not only single interactions but also triggers a cascade of alterations [15]. The ability to display a multitude of alterations renders it particularly suitable for the evaluation of combined exposure to toxicants [16]. Moreover, proteomics allows molecular fingerprinting of protein expression changes following the exposures to low levels of contaminants, where conventional methods of toxicology may not be sufficiently sensitive [17]. More importantly, perhaps, a set of proteins can potentially achieve higher accuracy and specificity than any single biomarker alone [18].

Oil is a complex mixture of hydrocarbons including PAHs which are strong carcinogens to humans and wildlife [19] and APs which were demonstrated to elicit oestrogen agonistic and/or antagonistic properties, thereby exerting hormone-modulating effects [20]. Even if these chemicals are rapidly diluted in the vicinity of offshore installations just after their discharge, the effluents may still exert low concentration effects [21], giving reason to evaluate whether these discharges may harm the biological resources in the sea.

As datasets from proteomic MS are typically characterised by large numbers of variables but relatively small sample sizes, untypical machine learning problems are encountered and supervised training of classifiers may be considered problematic [22]. In particular, overfitting of the multivariable models may seriously undermine the biological relevance of the sets of proteins constituting the classifier [23,24]. Owing to these concerns, the present study focuses on pre-processing and analysis of proteomic MS data generated with SELDI, thereby taking a refined and more conservative approach then reported previously [5,8,9]. To evaluate the developed protocol for data processing and classifier generation, different datasets derived from a 21-day exposure of Blue mussels in a laboratory flow-through system to crude oil with and without a spike of PAHs and APs were used. We included feature selection based on univariate statistical methods prior to decision tree classification. For final evaluation, a minimum variation threshold for the definite peaks was considered in order to advance biological relevance and robustness of the protein expression signatures [25,26].

## Results

### SELDI profiling of gill proteins

A panel of gill-spectra (n = 51 for C, n = 66 for oil and n = 55 for sO; note: the discrepancy between the final number of spectra analysed and the number of animals sampled in total results from the removal of low quality spectra prior to analysis; see material and methods section) yielded 55 peaks with differential expression. Statistical analysis revealed 20 of these to be significantly altered, with levels of differential expression below 50%. Just one peak (*m/z *4755) displayed approximately two-fold alteration, albeit with rather high variability and hence low significance (0.05 ≥ *p *> 0.01). Of the 20 peaks showing significantly altered peak intensities, only six were classified highly significant (*p *< 0.001). The most prominent peaks, combining high statistical significance with at least 50 % change in expression were *m/z *6696 found in oil and sO as well as *m/z *9661 and *m/z *14 847 in oil only, all of which were down-regulated (Table 1A). Remarkably, the effect of oil exposure on gills tended to be more pronounced than that of sO, as generally more peaks were found at a highly significant level and the expression changes were slightly stronger (data not shown). In addition, eight peaks were unique to oil exposure (data not shown), amongst them the one at *m/z *9661.

**Table 1:** Differentially expressed peptides/proteins from gills (A) and digestive gland (DG, B) of Blue mussels obtained from controls (C) and 21d exposures to 0.5 mg/L crude oil (oil) and 0.5 mg/L crude oil spiked with a mixture of APs and PAHs (0.1 mg/L; sO); mean intensities (arbitrary values) and SD of controls (Gills, n = 51; DG, n = 74), oil (Gills, n = 66; DG, n = 71) and sO (Gills, n = 55; DG, n = 69). Expression changes relative to the controls are significantly different at a level of *p *< 0.001 (one-way ANOVA for C *vs*. oil *vs*. sO) at least for one of the exposures. Peaks are listed in decreasing order of their respective *p *values from top to bottom.

Peak [m/z]	Mean intensity		
	**C**	**oil**	**sO**
**A – *Gills***			

Down-regulation			
14847	**1.01 **± *0.32*	**0.61 **± *0.26*	**0.81 **± *0.31*
6696	**2.76 **± *1.19*	**1.86 **± *1.34*	**1.64 **± *1.33*
9661	**8.11 **± *3.82*	**5.35 **± *3.63*	**8.35 **± *5.06*

**B – *DG***			

Up-regulation			
7811	**0.83 **± *0.74*	**1.46 **± *1.22*	**2.76 **± *1.84*
16196	**0.75 **± *1.20*	**1.23 **± *1.46*	**2.18 **± *2.06*
4744	**0.43 **± *1.05*	**0.65 **± *1.60*	**1.37 **± *1.80*
3795	**0.84 **± *1.42*	**1.33 **± *2.19*	**2.64 **± *3.39*
3984	**1.04 **± *2.03*	**1.04 **± *1.97*	**2.67 **± *3.05*
4108	**0.49 **± *1.36*	**0.44 **± *1.32*	**1.34 **± *1.95*
3552	**0.69 **± *2.00*	**0.54 **± *1.34*	**1.67 **± *2.66*
28764	**0.76 **± *0.7*7	**1.78 **± *1.45*	**1.76 **± *1.23*
52306	**0.34 **± *0.25*	**0.72 **± *0.73*	**0.46 **± *0.44*

Down-regulation			
9172	**3.78 **± *4.26*	**2.46 **± *2.21*	**1.01 **± *0.86*
18250	**0.47 **± *0.40*	**0.23 **± *0.26*	**0.15 **± *0.17*
7410	**1.09 **± *0.59*	**0.74 **± *0.57*	**0.55 **± *0.32*

### SELDI profiling of digestive gland proteins

From the panel of DG-spectra (n = 74 for C, n = 71 for oil and n = 69 for sO), 88 peaks were found to be differentially expressed. Of the 49 peaks showing significantly altered peak intensities, more than 60 % were highly significant at a level of *p *< 0.001 and the expression levels of almost 30 % were altered more than two-fold, the majority of which were up-regulated (Table 1B). In terms of significance and level of expression change, the peaks at *m/z *7811 and *m/z *9172 should be highlighted, especially for their response to additional PAHs and APs.

**Figure 1:**
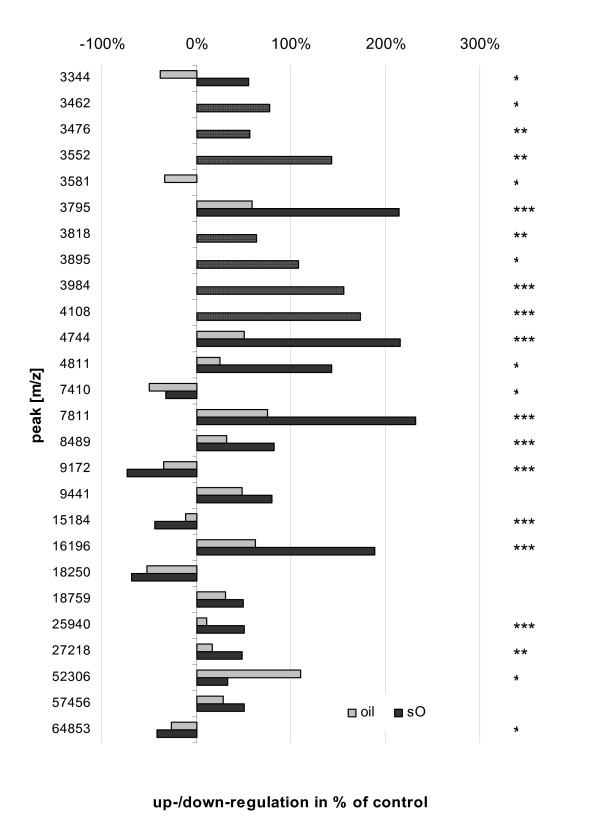
Relative expression changes of differentially expressed peptides and proteins in the digestive gland of Blue mussels following 21d exposure to 0.5 mg/L crude oil (oil) and 0.5 mg/L crude oil spiked with a mixture of APs and PAHs (0.1 mg/L; sO) in % of control intensity; *, significantly different at 0.05 ≥ *p *≥ 0.01, **, at 0.01 ≥ *p *≥ 0.001, ***, at *p *< 0.001. Dotted bars indicate differential expression occurring in one of two exposures only.

In general, increases or decreases of expression detected in both types of exposure behaved consistently, commonly being less pronounced in the oil-exposure. Evidently, spiking crude oil with APs and PAHs augmented responses compared to oil alone in a somewhat 'dose dependent' manner (Figure 1). Moreover, some peaks appeared to be altered solely in sO-exposure (dotted bars in Figure 1). Spike-related intensification of differential protein expression was also characterised by higher levels of significance for certain sO-peaks (data not shown).

### SELDI profiling of sex-dependent responses

To obtain information on differential expression with respect to the gender of the mussels, the datasets were split into six groups, a male and a female one for the controls and the two exposures respectively. Consequently, in the face of high inter-individual variability, the statistics were weakened. This was reflected in fewer percentages of highly significant peaks (*p *< 0.001; gills: 14 % for females, 21 % for males; DG: 27 % for females, 42 % for males). The majority of these peaks were already displayed in the overall analysis in which gender information was not included. For example, the most prominent peaks for DG, *m/z *7811 and *m/z *9172 are found to be highly significant in males and in females with a strong degree of up- or down-regulation. However, by splitting the data, additional information on these peaks could be acquired, as for instance, the peak at *m/z *7811 obviously responded more strongly in females than in males: in oil-female, it shows a two-fold increase (*p *< 0.001) compared to a non-significant 1.5 fold in the oil-male as well as a 4.5 fold increase in sO-female compared to 2.5 fold in sO-male, both at *p *< 0.001. Similarly, the peak at *m/z *18 250 shows a significant 3 fold down-regulation for the females of the oil exposure plus a highly significant 5 fold down-regulation for the sO-females but this is less pronounced in males with a 1.5 fold decrease for oil (*p *≤ 0.05) and 2.5 fold decrease for sO (*p *< 0.001). Accordingly, these incidences characterise the sex-differentiated variations of a general response.

**Figure 2:**
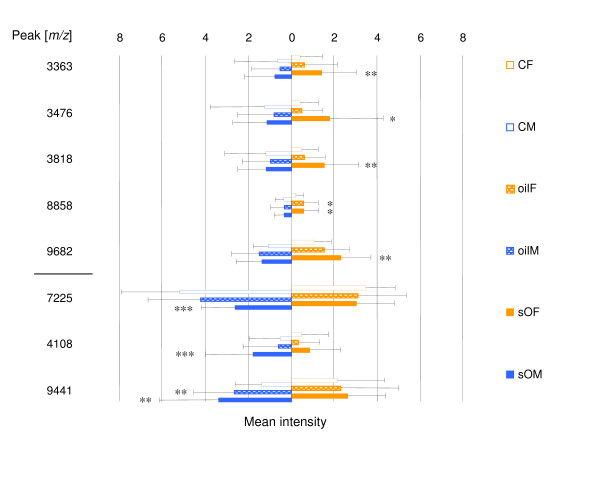
Histogram of sex dependent expression changes (intensities as arbitrary units) obtained for digestive gland of *M. edulis *upon 21d exposure to 0.5 mg/L crude oil (oil) and 0.5 mg/L crude oil spiked with a mixture of APs and PAHs (0.1 mg/L; sO); CF = control females, CM = control males, sOF = spiked oil females, sOM = spiked oil males, oilF = oil females, oilM = oil females; males are in blue and females in orange; the upper five represent female-specific and lower three male-specific peaks. Significantly different at 0.05 ≥ *p *≥ 0.01, **, at 0.01 ≥ *p *≥ 0.001, ***, at *p *< 0.001.

To extract truly male- or female-specific responses, the univariate statistics carried out for males and females separately have been 'subtracted' from each other, in order to keep only peaks appearing in one of the genders. In DG, where most of the sex-specific alterations were obtained, 22 peaks showing differential expression were unique to females and 20 to males. Regarding the levels of differential expression, 55 % of these male-specific peaks were altered more than two-fold compared to only 36 % in females. Eventually, the sex-specific peaks were crosschecked for similar but non-significant differential expression, and if such occurred, those responses were regarded less likely to be genuinely sex-specific and thus the peaks were excluded. Figure 2 shows the most definite peaks for DG, which were attributed to a clear sex-specific response; notably they were all found to be in the peptide range. Another five peptides represented some kind of sex-differentiated response in which up-regulation was found in one gender and down-regulation in the other, four of which were significantly altered only in males. The majority of these alterations were triggered by spiking the crude oil with additional APs and PAHs.

**Figure 3:**
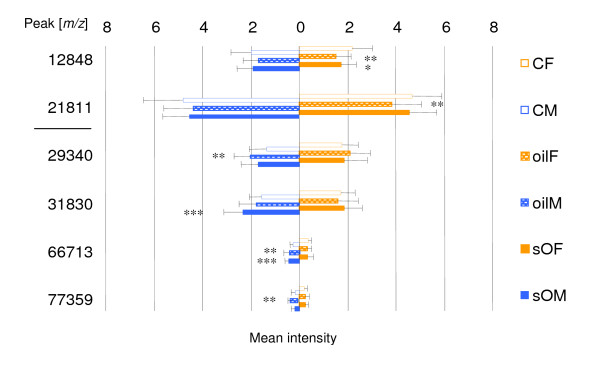
Histogram of sex dependent expression changes (intensities as arbitrary units) obtained for gills of *M. edulis *upon 21d exposure to 0.5 mg/L crude oil (oil) and 0.5 mg/L crude oil spiked with a mixture of APs and PAHs (0.1 mg/L; sO); CF = control females, CM = control males, sOF = spiked oil females, sOM = spiked oil males, oilF = oil females, oilM = oil females; males are depicted as blue and females as orange; the upper two represent female-specific and lower four male-specific peaks. Significantly different at 0.05 ≥ *p *≥ 0.01, **, at 0.01 ≥ *p *≥ 0.001, ***, at *p *< 0.001.

In addition, the datasets for gills were examined for sex-specific responses. Although this tissue was expected to be less prone to respond in a sex-specific manner, some proteins were revealed; the most important ones are depicted in Figure 3. These proteins were predominantly found in males, the majority of which exceeded 10 kDa. There was but one remarkable exception: more detailed analysis of the data revealed a peptide of *m/z *4185 that was practically absent in the controls and induced in oil (~17-fold) and sO (~10-fold), although only significant in the latter at a level of *p *≤ 0.05 due to an enormous variability as indicated by a high SD.

### Classifiers

The above data analysis was complemented by generating classification models using pattern recognition software based on CART to validate the predictive value of the differentially expressed proteins. In principal, the models on the gill data required more nodes in the decision tree associated with higher error costs to carry out the classification to a satisfactory degree than the ones for the DG data. This is likely to diminish their predictive reliability and accounts for the absence of robustness as was confirmed by comparing the estimated classification success on the basis of random cross-validation of 10% of the LS with the actual classification success as validated with the TS. The two best models for gills predicted a relatively good classification with 73 % of the oil-samples correctly attributed, 81 % of the sO-samples and 88 % for the controls each. Only the sO-model for DG assumed to perform a better classification of 91 %. Still, the overall classification success for gills, when tested with unknown samples, turned out not to be as estimated and less good than for DG (Table 2). For example, with the gill-TS, merely 50 % of the sO-samples could be assigned correctly and for oil-exposure, only 60 % of the controls were recognised (Table 2A). In other words, both models generated for gills showed sufficient classification success only for one of two groups. On the contrary, the models for DG yielded relatively good classification success for the controls and sO-samples; just the oil samples showed less than 70 % correct classification (Table 2B). However, these models also displayed significant discrepancy between estimated and actual classification successes (maximum 11 percentage points), although not in the same extent as for the gills (maximum 31 percentage points).

**Table 2:** Prediction success of the classifiers for gills (A) and digestive gland (B) from the Blue mussel for pairwise comparison of controls (C) with either of the two different exposures to i) 0.5 mg/L crude oil (oil) and ii) 0.5 mg/L crude oil spiked with a mixture of APs and PAHs (0.1 mg/L; sO) for 21 days as validated with an independent sample set not used in model construction. Sample size for learning and testing set, amount of samples attributed to each class, individual and overall classification success in % of testing set is presented.

**Prediction success**							
**A **– *Gills*							
**Class**	**Learning**	**Testing**	**%**	**C**	**oil**	**sO**	**Tot %**

**C**	34	17	65	11	6		72
**oil**	44	22	77	5	17		
**C**	34	17	82	14		3	66
**sO**	37	18	50	9		9	

**B **– *DG*							

**Class**	**Learning**	**Testing**	**%**	**C**	**oil**	**sO**	**Tot %**

**C**	48	26	85	22	4		76
**oil**	47	24	67	7	17		
**C**	48	26	77	20		6	80
**sO**	45	24	83	4		20	

The models for gills and for DG did well reflect the significance of certain peaks as ranked by univariate statistics and by their levels of up- or down-regulation (Table 1 and Table 3). Since the models for gills and for DG were generated for two groups only (C *vs*. oil plus C *vs*. sO), the most important peptides and proteins as listed in Table 1 were not necessarily represented in both models. Whenever this was the case, they did not exactly display the same discriminatory power for classification of oil and sO samples respectively (Table 3A and 3B). Attempts to generate classifiers with three groups including controls and both of the exposures failed. Those classifiers were unable to effectively distinguish controls from exposures and thus were omitted. If taken together, though, most of the prominent peaks determined by Kruskal-Wallis One-way ANOVA can be retrieved in either of the two classifiers for oil and sO.

**Table 3:** Peak constituents of the optimal classification models for gills (A) and digestive gland (B) of Blue mussels exposed to 0.5 mg/L crude oil (oil) and 0.5 mg/L crude oil spiked with a mixture of APs and PAHs (0.1 mg/L; sO) for 21 days. The score equals the discriminatory weight of the variable within the classifier. A pre-selection of highly significant peaks (*p *< 0.001) has been carried out prior to model construction.

**A **– *Gills*			
oil *vs*. C		sO *vs*. C	
Peak [m/z]	Score [%]	Peak [m/z]	Score [%]
	
14847	100.00	6696	100.00
9661	86.57	28017	45.98
6696	65.34	40456	35.26
44667	54.41	14847	27.95
28017	52.31	110694	19.99
40456	31.73	4755	18.53
12848	12.86	31830	17.12
20316	10.60	6103	16.51
29340	9.09	88989	14.53
		44667	12.89

**B **– *DG*			

oil *vs*. C		sO *vs*. C	
Peak [m/z]	Score [%]	Peak [m/z]	Score [%]
	
16413	100.00	7811	100.00
28764	89.59	9172	55.46
18250	77.33	18250	54.69
12481	54.11	7410	47.04
7410	48.11	4744	40.56
		8489	21.94
		3984	20.99
		16196	13.41
		12481	11.73
		28764	8.17

## Discussion

By using one surface-chemistry for gills and DG, which were assumed to be most affected targets in mussels, it was possible to compare differences in the response of two dissimilar tissues to oil exposure. At first, it was found that both tissues differ remarkably in number, type and amplitude of differentially expressed peptide and protein peaks. Such differences may have an important influence on the performance of classifier generation and could render certain datasets unsuitable for tree-structured analysis (see below). In consequence, the choice of tissue investigated by MS proteomic profiling for the construction of decision tree models is not trivial. It cannot be excluded that some of this observed difference may be attributed to minor alterations in the protein extraction procedure, which were necessary due to the high amounts of lipids in the DG. However, we do not believe these were the most important factors in causing overall differences in the profiles. The DG represents a major site of synthesis and detoxification [27,28]. With respect to the complexity of its functions, it is not surprising that a higher amount of differentially expressed peptides and proteins in response to toxicant exposure could be obtained.

Nonetheless, classifiers have been generated for both gill and DG data sets by subjecting all proteins and peptides with highly significant expression changes to pattern recognition analysis, an artificial learning algorithm that generates supervised classifiers. Generally spoken, the dual aim of carrying out class prediction is i) to evaluate the discriminatory power of differentially expressed proteins and ii) to identify a so-called protein expression signature indicative of the type of exposure. This is based on the following suppositions: first, it is expected that the higher the discriminatory power of a variable, the more relevant it should be as a potential biomarker; second, as suggested earlier [1], the set of proteins itself may serve as a biomarker (thus also-called 'biomarker pattern') as it is able to distinguish between control and exposed animals. The concept, that a set of multiple marker proteins acting in concert produces better classifiers containing a higher level of discriminatory power than could be obtained by a single biomarker alone has been widely acknowledged [18,23].

Unlike the hypothesis driven approach, which investigates known molecules, proteomics may reveal associations between proteins and exposure to contaminants that have not been described previously. However, the identification of several proteins in question is a laborious and time-consuming operation. Even worse, it is derogated by the lack of database information on non-model organisms [29,30], in particular for many invertebrate species such as mussels, which to date are poorly characterised at the genome and proteome level [31]. Previous attempts to identify the constituents of expression signatures in bivalves obtained from the exposure to pollutants documented difficulties in identifying key proteins [3,6,7,10]. Some evidence suggests that a bias towards cytoskeletal proteins emerged [3,6,10,32], presumably due to their relative abundance and prevalence in databases [3]. Therefore, even if informative protein markers can be extracted from proteomic datasets, their identification may remain elusive. While it will definitely aid understanding of the mechanistic role and hence improve the diagnostic reliability [1], it is not imperative to know the identity of an effective biomarker of exposure, given that it would constantly appear under certain conditions [2]. Taking this idea further, a set of proteins and peptides, specific to a particular stressor would constitute the diagnostic marker, independent of their identity. Biomarker pattern recognition based on tree-structured analysis of large-scale proteomic MS data certainly represents a powerful approach to achieve this goal.

It should also be noted that even though the correlation of proteomic alterations with biochemical pathways would render proteomic assays more powerful and should eventually be aspired where possible, in many cases the significance of expression changes is not validated prior to protein identification. Indeed protein identification often represents a means of evaluating such significance by attempting to reveal a biologically plausible function, which in turn would demonstrate their involvement in responses related to pollution exposure [3,6,7,10]. Oberemm *et al*. [17], for instance, could confirm the differentially expressed proteins of the thymus tissue of marmoset exposed to TCDD to be related to immune function, which is particularly affected by this substance. In ecotoxicology, however, where non-model organisms are frequently employed for monitoring purposes, such confirmation often fails as database matches cannot be obtained for the key proteins of the exposure derived expression signatures [3,6]. It has been suggested, though discussed critically, that the evaluation of proteomic patterns from MS proteomic profiling can proceed independently from the identities of their proteins [18,33-35] and that classification algorithms would represent a means to extract informative proteins from such data [11,13].

In comparison to earlier studies [5,8,9], however, we experienced limitations to the generation of classifiers resulting in lower sensitivity and specificity of the classification success. Bjørnstad *et al*. [8] presented discrimination models using mussel haemolymph, which were able to classify oil-exposed mussels with more than 90 % accuracy. Yet, statistical constraints resulting from too many variables in combination with too few samples may have impaired classifier construction and resulted in overfitting of the models [22,24]. In general, few sample numbers per class make it easy to produce seemingly robust classifiers that give excellent results for both LS and TS [13,23]. Accordingly, the reliability of such 'optimal' models may be illusory, notwithstanding their very good classification success. In contrast, when reducing the dimensionality of the dataset prior to classifier construction, the highest classification success was 80% for the DG dataset and sO exposure. To improve the extraction of biologically relevant predictors and to guard against overfitting, we included feature selection as proposed by Levner [22]. Using univariate statistical tests, which rank the variables according to their significance, we decided to only integrate highly significant peaks (*p *< 0.001) into the models, thereby also minimising the risk of including falsely differentiating peaks. In addition, we ranked the robustness of the models before maximum classification success by considering only those decision trees with the lowest misclassification costs (*i.e*. low error rates). Consequently, the poorer classification success as compared to the previous studies is owed to improved and more prudent data processing.

From the comparison of gill and DG datasets, as well as from the investigation of proteins and peptides with sex-specific differential expression, two major conclusions could be inferred: (1) As the overall expression changes observed with gills resulted in fewer peaks with a significance level of *p *< 0.001 (which, in addition, showed notably lower expression changes than those of DG) the gill dataset performed relatively poor in the actual classification of unknown samples. Besides, none of the *m/z *values contained in the classifier reaches a twofold expression change. Conversely, the DG dataset was able to construct better decision rules, which were more robust, resulting in a higher actual classification success. It can thus be concluded, that a sufficient number of peaks statistically corroborated, possibly combined with minimum amplitudes of differential expression, is required to enhance the discriminatory power and reliability of a biomarker pattern. Not all datasets will be equally suitable. (2) Splitting the datasets into male and female for each of the tissue, plus the requirement to separate them into LS and independent TS rendered them too small to result in appropriate class prediction. This clearly emphasises the importance of sufficient biological replicates and hence large enough sample sets. Incorporation of gender information into the entire dataset, did not give any satisfactory results as to specific sex-dependent proteins and peptides. Eventually, sex-specifically altered proteins and peptides could only be deduced from statistical significances, which as well have been substantially weakened by reduced sample numbers. Statistical significance by itself, however, does not provide any indication of the diagnostic value of those peaks. Concerning the number of biological replicates, it should be noted that the tree-growing methodology is data intensive. Thus, even though reducing the number of variables for the input space (*i.e. m/z *peaks) by introducing a filter, eventually much larger datasets would be needed for classifier validation.

Similar ecotoxicological case-control studies have been analysed recently, either by SELDI or 2-DE [6,8-10]. Some of the complex data resulting from those analyses have then been subjected to various data mining techniques such as principal component analysis, hierarchical clustering, non-metric multidimensional scaling and CART, resulting in the conclusion that a set of proteins specific or indicative of the treatment has been obtained, some of which were identified as related to the metabolism of xenobiotics [7,10]. The strength of those conclusions largely depends on the study design as well as on data-acquisition and data-mining methods. With the ebullient expectations of applying proteomics to ecotoxicology, the consideration of factors compromising these findings may have been insufficient. For instance, constitutive proteomic expression changes are not well investigated and expected to vary widely with differences in age, diet or developmental stage. Accordingly, the changes due to toxicant exposure may not be greater than the noise of protein expression variability [36]. Secondary experimental effects, such as reduced food intake and energy deprivation, may also account for the observed proteomic changes. Many of the alterations are likely to be of a more general nature and would become manifested with quite different types of pollutant-derived stress as well as with otherwise adverse environmental conditions. This might also be one of the reasons for repeatedly identifying ubiquitous genes and proteins in varying experiments, such as cytoskeletal proteins (actin, mysion, tropomysion, tubulin [3,6,10]), malate dehydrogenase [6,37], glutathione S-transferase [38,10] or proteins associated with physiological pathways of respiration (*e.g*. carbonic anhydrase [39,10]) and oxidative stress (variants of superoxide dismutase [7,10,38]). Experimental repeatability and reproducibility also represent important information that needs to be investigated and monitored more thoroughly [40]. Eventually, data analysis protocols including data processing steps (*i.e*. filtering of noise, data normalisation, peak/spot matching, peak/spot detection and quantification) as well as the data-mining methodology can be largely varied. This in turn will have a significant impact on the protein patterns generated.

Once established in the laboratory where natural variation is reduced, as for any other biomarker, an expression signature has to be confirmed in field situations, most likely with varying sources and compositions of chemicals as it is typically the case for mixture pollution as well as involving different populations. Natural biotic and abiotic conditions will influence the differential expression of proteins at the various sites. These confounding factors already complicate the application of many biomarkers in the field. As an example, Arts *et al*. [41] have reported very high variability of heat shock protein levels and discussed their suitability for field studies. With mussels, tidal, diurnal and seasonal effects are likely to change baseline proteomic expressions similar to the expression changes observed with specific proteins [42-44]. Even though a pattern of multiple marker proteins is expected to have a higher predictive value than a single biomarker [18], in ecotoxicology the problematic of multifactorial action of confounding factors along with possible non-monotonous concentration response relationships [41] may be actually magnified in the multivariate proteomic profiles. On the other hand, supervised learning methods could possibly ignore non-informative biological variance in the datasets and segregate unspecific from specific responses, thereby selecting those variables only that are able to indicate the particular character of an exposure. Additionally, randomisation and matching of potential confounding factors (age, sex, etc.) prior to data analysis would be likely to prevent biases in the obtained results [36]. As such, the proteomics based biomarker pattern, could integrate specific as well as secondary effects, all of which are part of the organisms' combat against toxic action.

Despite the potential of proteomic patterns as sensitive markers that comprise multiple molecular endpoints with high discriminatory and ideally explanatory power, a careful approach has to be taken in each step of proteomic profiling, biomarker discovery and validation. Errors in study design and execution can lead to misleading results, especially when exploiting the vast datasets using complex multivariate analyses [13]. It then has to be demonstrated whether these patterns are robust enough to be retrieved in the face of biological variability and if they can be related causally or at least linked statistically to higher levels of biological organisation [45], before proteomics may be involved into risk assessment.

## Conclusion

Machine learning and classification algorithms may represent a powerful means to extract relevant information from the large data sets obtained by MS proteomics. However, supervised training of classifiers is prone to overfitting, resulting in excellent classification success and thus has to be conducted with caution. Moreover, CART performs better with larger sample sizes, which could be processed by SELDI due to its high-throughput capacities and the possibilities for standardisation and automatisation of procedures, but may not be available from sample collection. Consequently, the optimal strategy to screen proteomics MS data from similar ecotoxicological studies has yet to be elaborated.

## Methods

### Organisms

Adult *M. edulis *were collected in November 2002 along the Førlandsfjord nearby Stavanger, Norway. Norwegian authorities had previously classified the Fjord as non-contaminated. The animals were transferred to the laboratory and placed in 123 L tanks per group with a flow rate of 3 L seawater per min. For the continuous flow-through system, natural fjord water of 4°C was taken from a depth of 80 m in the water column [46]. Each tank, one per exposure and control, contained 250 individuals with a size range of 6.2 to 9.0 cm. The mussels were fed every second day with an algae mixture consisting of *Rhodomonas sp*. and *Isochrisis sp*. Acclimation to laboratory conditions lasted for 13 days prior to starting the experiment.

### Exposure

The Blue mussels were allocated into three groups: i) control mussels (C), ii) mussels exposed to 0.5 mg/L of crude oil (oil) and iii) mussels exposed to 0.5 mg/L crude oil spiked with a mixture of 0.1 mg/L combined APs and PAHs (sO) as nominal concentrations for 21 days. Prior to the insertion of the animals, the surfaces of the exposure tanks have been pre-absorbed with the exposure media for three days. A minimum of 30 males and 30 females were sampled for each group. The determination of the gender was carried out by smeared probes of the gonads observed by light microscopy.

North Sea crude oil (Stratfjord B) was dispersed into seawater mechanically by a mixing valve and a Dispax^® ^rotator, running at a speed of 10 000 rpm. The droplet size was monitored frequently by a Coulter^® ^II particle size analyser. Oil concentrations in the water were calculated from the estimated particle size and number [47]. The spike of APs + PAHs represented an additional boost of low molecular size PAHs (Naphthalene plus C1–C3 alkyl homologues, Fluorene, Phenanthrene plus C1–C2 alkyl homologues, Dibenzothiophene plus C1–C2 alkyl homologues; sumPAH: 0.018 mg/L]) and the most common APs found in produced water (p-cresol, m-ethylphenol, 3,5-dimethylphenol, 2,4,6-trimethylphenol, 2-tert-butylphenol, 3-tert-butylphenol, 4-n-butylphenol, 4-pentylphenol; sumAPs: 0.082 mg/L). Both the APs and the PAHs were based upon previous studies of their composition in PW discharges and monitored by HPLC analysis [47,48]. More detailed analysis information can be obtained from Bjørnstadt *et al*. [8]. A general description of the experimental design is given in Sundt *et al*. [49].

### Protein extraction and chip preparation

Gills and DG were dissected from live mussels, snap frozen and stored at -80°C until further processing. Previous method optimisation revealed strong anionic exchange chip surface to yield in a maximum number of peaks with both tissue types und thus represented the surface chemistry of choice for proteomic expression profiling [5]. Although not assessed in this study, reproducibility has been evaluated prior to method implementation and was found to be satisfactory. Coefficients of variance for SELDI applications have been reported to be reasonably below 20% indicating that SELDI experiments are consistent at the level of common proteins expressed [8,9,50]. However, according to Listgarten and Emili [14] these are no absolute indicators of quality as coefficients of variance are less informative when feature detection is part of the analytical process. In a quality assessment study conducted by Hong *et al*. [50] systematic variability across spot position as well as across chips or plates has not been detected, demonstrating good reproducibility of SELDI experiments.

Protein extraction and chip preparation were carried out as described previously [5]. Briefly, tissues were homogenised with 50 mM Tris, pH 7.6, 150 mM NaCl, 0.1% Triton X-100, 10 mM DTT (1:4 w/v for gills and 1:8 w/v for DG) on ice and centrifuged three times for 20 min at 20 000 *g*, 4°C. To avoid protein breakdown during preparation, a protease inhibitor cocktail (Sigma-Aldrich) was added to the samples. Because lipids generally disturb the binding of proteins to the chip surface, they were absorbed in the lipid rich DG homogenates by Liposorp™ (Calbiochem; 1.5:1 v/v). Total protein content of the supernatant was determined according to the Bradford method [51]. Protein concentration was adjusted to 1 mg/mL prior to sample dilution with binding buffer (50 mM sodium acetate, 0.1% Triton X-100, pH 5.5) resulting in a final protein concentration of 1 μg/mL. Per spot, 20 μg of protein was incubated overnight at 4°C in a 96-well bioprocessor (Ciphergen Biosystems Inc.) on a platform shaker. Three washes with binding buffer devoid of Triton X-100 and two quick washes with ultra pure H_2_O subsequently removed unspecific bound proteins. Sinapic acid resolved in 50% ACN/0.1% TFA was applied as a matrix. The arrays were processed according to an automated data collection protocol in a PBS-II protein chip MS reader. Mass accuracy was calibrated externally using the 'All-in-1' molecular mass standard (Ciphergen Biosystems Inc.).

### Spectral processing

The raw intensity data were pre-processed prior to subsequent protein expression profiling using ProteinChip^® ^software version 3.1.1 (Ciphergen Biosystems Inc.). All spectra of the three different groups were assembled and examined for two separate regions on account of different noise levels [52,53]. Normalisation of peak intensities to total ion current was from *m/z *3000 to 20 000 for the low molecular weight area and from *m/z *20 000 to 100% of the spectrum size for the high molecular weight range. Despite generally good overall consistency among spectra for SELDI experiments, the reproducibility does not define the quality of individual spectra [50]. Hence, low quality spectra need to be identified [40,50] by defining outliers and should be removed prior to data analyses. This was carried out by using the quartile method on the basis of calculated normalisation factors. For mass-normalisation, identical peaks were utilised in the low and high molecular weight area to assure the compatibility of peak detection. Gill spectra were internally calibrated with three peaks found in all of the spectra (*m/z *9242, 16 284 and 40 454). For the calibration of DG spectra four prominent peaks could be utilised (*m/z *7206, 9538, 21 643 and 35 687). Peak detection was similar for low and high molecular weight ranges, except for the cluster mass window which was 0.5 % up to *m/z *20 000 and 1 % for the rest of the spectral region. The S/N was set at three for the first and two for the second pass with baseline subtraction on. Peaks were required to be present in a minimum of all spectra, equivalent to approximately two-thirds of the spectra of one group (*i.e*. 20 % when peaks of C, oil and sO where clustered or 12 % if the spectra where additionally divided into male and female groups). Estimated peaks were added by the software.

### Data analysis

For an initial statistical evaluation of the two datasets, nonparametric Kruskal-Wallis One-way ANOVA was performed on all profiles (C *vs*. oil *vs*. sO). For the identification of sex-specific peaks, the datasets were split into the male and female fraction of each group. A protein or peptide was considered to be differentially expressed if a statistically significant alteration in its intensity was observed when compared to the control group. The overall significance level was set at 5 % and the variables were ranked according to their statistical significance with three levels of significance (0.05 ≥ *p *> 0.01; 0.01 ≥ *p *≥ 0.001 and *p *< 0.001).

In view of the variance of peak intensities between individual spectra, and the prevalent low expression changes, we focussed on the highest level of significance (*p *< 0.001) for the most informative and robust variables [54]. According to the range of expression changes found in this and earlier studies with SELDI and Blue mussels [5], the ones with more than two-fold up- or-down-regulation were considered more likely to be steadily discriminable from random variation [55]. This empirical cut-off value, corresponding to a log ratio with an absolute value bigger than 0.3, is widely found in literature [*e.g*. [25,55]] and has been statistically confirmed by Sabatti *et al*. [26].

To determine the diagnostic value of marker candidates, the LS was subjected to supervised classification analysis using BiomarkerPattern™ software, version 4.01 (Ciphergen Biosystems Inc.). BiomarkerPattern™ software is an implementation of CART, a nonparametric statistical procedure based on the binary recursive partitioning algorithm introduced by Breiman *et al*. [56], with cost-complexity pruning by 10-fold cross-validation. Details of CART analysis have been described elsewhere [13], [57]. Briefly, CART begins with a root node and, through a series of yes/no questions, generates descendent nodes until final classification is reached or further splitting is terminated (*e.g*. too few cases). Each split separates a parent node into exactly two child nodes using one rule at a time. Here, the splitting rules depend on the normalised intensity levels of the *m/z *values obtained from the SELDI protein expression profile. Once maximal trees are grown, smaller sub-trees are generated by pruning away the branches of the maximal tree and the best tree is determined by testing for error rates (*i.e*. costs of misclassification). The intuitive presentation of decision rules in a tree-structured form facilitates the interpretation and applicability of the obtained classifiers.

For the construction of decision tree models, the datasets were divided randomly into a LS which comprised about two thirds of the spectra of the respective groups and a TS consisting of the remaining third (Table 2). Sample statistics, defining the input variables for classifier generation were performed on the LS for C *vs*. oil and C *vs*. sO by Mann-Whitney U-test. In order to reduce the dimensionality for model construction and to obviate false positive discoveries of potentially discriminating variables [13] the input matrix was restricted to normalised intensity levels with *p *< 0.001.

Different models were constructed by varying user-defined criteria (*e.g*. Gini power), whereby CART selects the variables in an independent manner for each model. Optimal model selection was carried out by the analyst, not merely for maximum predictive success but obligatory involves low 'error costs' and few decision nodes within the tree-building algorithm in order to ascertain the robustness of the model. Moreover, estimated classification success was supposed not to differ too much between the respective groups. Subsequently, the chosen models were independently tested with the TS, a set of spectra not involved in the generation of the classifier. This was done to verify whether the actual and estimated classification success would be in good agreement, thus signifying the concluding criterion for applicable decision models.

In this study we have examined tree-structured classification with respect to two-class problems only. Theoretically, there is no limitation to the number of categories for classifier generation; also additional information, such as gender can be included. However, in our trials the outcome resulted in poor prediction success or did not provide any valid additional information; consequently those approaches were not included.

## Abbreviations

**ACN**, acetonitrile; **APs**, alkylated phenols; **C**, controls; **CART**, classification and regression trees, **2-DE**, two-dimensional gel electrophoresis; **DG**, digestive gland; **DTT**, dithiotreitol; **HPLC**, high-performance liquid chromatography; **LS**, learning set; **MS**, mass-spectrometry, **m/z**, mass-to-charge ratio; **oil**, oil exposure; **PAHs**, polycyclic aromatic hydrocarbons; **PW**, produced water; **SD**, standard deviation; **SELDI**, surface-enhanced laser desorption/ionisation time-of-flight mass-spectrometry; **S/N**, signal-to-noise ratio; **sO**, spiked oil exposure; **TFA**, trifluoroacetic acid; **TCDD**, 2,3,7,8-Tetrachlorodibenzo-*p*-dioxin; **TS**, testing set

## Competing interests

The author(s) declare that they have no competing interests.

## Authors' contributions

OKA conceived the study, rose funding and participated in design and coordination of the experiments. TM and TK were involved in the experimental work; carried out sampling, sample preparation and SELDI-TOF mass spectrometry as well as data analyses. FL provided helpful suggestions and assisted in finalising the manuscript. TM and TK established the procedures of data processing and classifier generation. TM drafted the manuscript and TK wrote the final version.
